# Water production function and optimal irrigation schedule for rice (*Oryza* *sativa* L*.*) cultivation with drip irrigation under plastic film-mulched

**DOI:** 10.1038/s41598-022-20652-3

**Published:** 2022-10-14

**Authors:** Jinyu He, Bo Ma, Juncang Tian

**Affiliations:** 1 School of Civil and Hydraulic Engineering, Ningxia University, Ningxia, 750021 Yinchuan People’s Republic of China; 2 Engineering Technology Research Center of Water-Saving and Water Resource Regulation in Ningxia, Ningxia University, Ningxia, 750021 Yinchuan People’s Republic of China; 3 Engineering Research Center for Efficient Utilization of Modern Agricultural Water Resources in Arid Regions, Ministry of Education, Ningxia, 750021 Yinchuan People’s Republic of China

**Keywords:** Plant sciences, Environmental sciences, Hydrology

## Abstract

This study determined the Water Production Function (WPF) and Optimal Irrigation Schedule (OIS) for rice (*Oryza* *sativa* L*.*) cultivated with drip irrigation under plastic film. Six different field capacity levels were established, 100% (W1), 90% (W2), 80% (W3), 70% (W4) and 60% (W5). The results showed that, the rice growth and yields and quality were significantly affected by the different irrigation treatments. The rice height and yield decreased from W1to W4, the W2 is the highest yield. The lower the amount of irrigation water applied was, the higher the Irrigation Water Use Efficiency values were. A WPF model was established for this cropping system, and the water sensitivity indices calculated by the mathematical model showed that the crop water sensitivity decreased in the order booting stage > flowering stage > tillering stage > filling stage. Based on this result, the OIS determined by the dynamic solution of several models was as follows: the optimal irrigation levels were 750 m^3^ ha^−1^ in the tillering stage, 2125 m^3^ ha^−1^ in the jointing-booting stage, 1050 m^3^ ha^−1^ in the heading-flowering stage and 325 m^3^ ha^−1^ in the milk stage. The WPF and OIS developed in this study provide a theoretical basis for the implementation of rice cultivation with drip irrigation under plastic film in arid regions of China.

## Introduction

Irrigation water is becoming increasingly scarce and expensive^1^. Water availability is one of the most important ecological factors determining crop growth and development, and water deficit can strongly inhibit crop yields^2^. However, water shortages and severe water waste are two inconsistent aspects of current water resource usage worldwide^3^. For this reason, in areas of irrigated and dryland agriculture in north-western China, crop production and sustainable development are severely constrained by water limitations during the growing season^4^. Rice (*Oryza* *sativa* *L.*) is one of the world's most important food crops and provides food and nutrition to more than half of the world's population; it is particularly important for developing countries^5^. China is the world's largest producer and consumer of rice^6^. Traditional conventional paddy field planting has many challenges, such as high-water consumption, excessive chemical fertilizer and pesticide application, severe soil water pollution, high levels of pests and disease, high labour intensity, high inputs, and low economic benefits. For these reasons, rice production is facing many difficulties^7^. Since the 1990s, theoretical and applied research on water-saving and high-yield irrigation technology for rice has developed rapidly, and a variety of new technologies for water-saving rice irrigation have emerged^8^. Plastic film-mulched has clear effects on soil water retention and increases soil temperatures; the soil hardness and soil bulk density decrease after film-mulched, which benefits the rice root system^9^. The results of comparative experiments on rice film-mulched and traditional irrigation treatments showed that film-mulched could improve the light, temperature and humidity conditions in the field but could also lead to the occurrence of instantaneous extremely high temperatures. Plastic film-mulched had a significant water-saving effect on rice cultivation and could increase the seedling emergence rate, the number of tillers and effective panicles, promote microbial activity and nutrient decomposition, prevent soil erosion and compaction, and alleviate water stress in rice. The results of experiments on different mulching methods for dry-farming rice showed that covered cultivation can have complementary advantages: mulching reduces the soil moisture content, reduces water loss, improves the soil structure, increases nutrient accumulation, increases the percentage of effective tillers, advances the growth period, and prolongs the filling stage^10^. Therefore, addressing the seemingly contradictory goals of high yields and water savings is an important factor in promoting agricultural economic development by decreasing water resource use. The fundamental way to solve this problem is to establish a rice water production function model and then effectively control the rice irrigation system.

A crop water production function is the mathematical relationship between crop yield and water inputs or crop water consumption during crop growth and development^11^. It can be used to determine the effect of different levels of water stress on crops at different times^12^. It is also necessary for studying inadequate irrigation^13^. Crop water production functions will be different by crop, location, year, irrigation and agricultural management techniques and should generally be determined by irrigation experiments based on local conditions^14^. The relationship between water demand and yield has been studied by domestic and foreign scholars in different crops, such as wheat^15^, maize^16,17^, tomato^18^, and cotton^19–22^. Many experimental studies have been carried out, and different crop water production function models have been established according to the results^23^.

Studies on rice water production functions conducted in China and other parts of the world have focused mainly on the relationship between water demand and rice yield. However, few studies have been performed on the water production function of rice under drip irrigation. In this study, the water production function model of rice with drip irrigation under plastic film-mulched was cultivated in the arid area of Ningxia in China by using field experiments and analysis methods. The objective of this study was to investigate the relationship between rice water requirements and yield under soil water deficit conditions and to establish a water production function model for drip irrigation in the arid region of China.

## Material and methods

### Experimental site and climate data

Field experiments were carried out for 3 years in Xixia town (2018, 2019, 38° 26′ N, 106° 03′ E, altitude of 1123 m)) and Hongguang town (2020, 38° 42′ N, 106° 20′ E, altitude of 1105 m) in Yinchuan, Ningxia. This area is characterized by scarce precipitation (about 198 mm annually), strong evaporation (more than 2004 mm annually) The soil type in Xixia town is a light grey soil, and the soil type in Hongguang town is an irrigation silty soil. The physicochemical properties of the soil are shown in Table 1. The irrigation water was obtained from pumped wells next to the experimental sites, and the water was filtered before irrigation.

**Table 1:** Basic property of the tested soils in experiments (0–60 cm).

Field site	Organic matter (g kg^−1^)	Total nitrogen (g kg^−1^)	Available nitrogen (mg kg^−1^)	Available phosphorus (mg kg^−1^)	Available potassium (mg kg^−1^)	Soluble salts (g kg^−1^)	pH (H_2_O)	Soil density (g cm^−3^)	Field capacity (%)	Texture
Pingjibao	9.51	0.38	35.8	6.5	122.0	0.91	8.5	1.41	21.9	Medium loam
Hongguang	12.23	0.71	55.0	9.8	151.0	0.98	8.6	1.45	22.1	Heavy loam

### Experimental design

**Figure 1:**
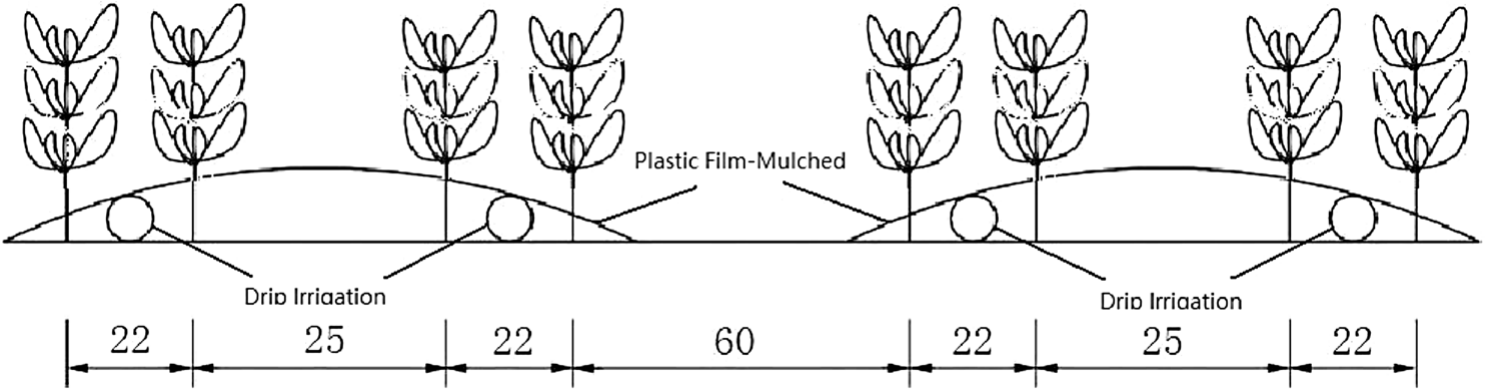
Planting pattern.

A total of 10 treatments were applied, and each treatment was repeated 3 times (Table 2). Each treatment consisted of a ridge that was 25 m long and 1.4 m wide. There were 4 rows in each ridge, and the row spacings were 22 cm, 25 cm and 22 cm. A drip belt was laid along every two rows under plastic film-mulched (Fig. 1). The planting distance was 13 cm, and approximately 30 seeds were planted in each hole. The experimental rice variety was 96D10. This variety is sown in early May, with emergence in mid-May and harvest at maturity in mid-October. To ensure an adequate emergence rate, the same amount of irrigation water was applied in all treatments in the seeding stage. At the tillering stage, the amount of irrigation began to be applied according to the experimental design. The water content of the soil was measured before irrigation to ensure that the soil moisture content stayed between the upper and lower limits for this study. Other management measures were the same as typical field practices in this area.

**Table 2:** Design scheme.

Treatments NO		Soil moisture (% of field capacity)			
		Tillering stage (%)	Booting stage (%)	Flowering stage (%)	Filling stage (%)
A	W1(CK)	100	100	100	100
	W2	90–100	90–100	90–100	90–100
	W3	80–90	80–90	80–90	80–90
	W4	70–80	70–80	70–80	70–80
	W5	60–70	60–70	60–70	60–70
	W6	50–60	50–60	50–60	50–60
B	W7	80–90	80–90	80–90	70–80
	W8	80–90	80–90	70–80	80–90
	W9	80–90	70–80	80–90	80–90
	W10	70–80	80–90	80–90	80–90

The actual soil moisture content of the field was determined as a percentage of field capacity(θ), reflecting the degree of soil water deficit. In group A, WI:100% of field capacity; W2:90–100% of field capacity; W3:80–90% of field capacity; W4:70–80% of field capacity; W5:60–70% of field capacity; and W6:50–60% of field capacity. In group B, soil moisture deficit conditions were applied at different growt stages. W7: a filling stage deficit (70–80% of field capacity); W8: a flowering stage deficit (70–80%); W9: a booting stage deficit (70–80%); and W10: a tillering stage deficit (70–80%). The relative soil water content during the other growth stages was maintained at 80–90% of field capacity.

The experiment included two groups (Table 2). In group A, the soil water deficit was tested, and six relative soil water levels were established (i.e., the actual soil moisture content of the field was determined as a percentage of field capacity(θ), reflecting the degree of soil water deficit): ① 100% of field capacity; ② 90–100% of field capacity; ③ 80–90% of field capacity; ④ 70–80% of field capacity; ⑤ 60–70% of field capacity; and ⑥ 50–60% of field capacity. In group B, soil moisture deficit conditions were applied at different growth stages. Four treatments were implemented: ⑦ a filling stage deficit (70–80% of field capacity); ⑧ a flowering stage deficit (70–80%); ⑨ a booting stage deficit (70–80%); and ⑩ a tillering stage deficit (70–80%). The relative soil water content during the other growth stages was maintained at 80–90% of field capacity. A TDR sensor was used to dynamically monitor the soil water content to regulate the irrigation amount from the tillering stage to the filling stage and maintain the specific water content of the soil in the different growth stages.

Uniform fertilization techniques were applied in the experimental plots. The basal fertilizer amounts applied at sowing included urea (N46%), 160 kg ha^−1^; diammonium phosphate (18-46-0), 160 kg ha^−1^; and compound fertilizer (15-15-15), 180 kg ha^−1^. During the growth period, urea was top-dressed through drip irrigation 10 times, for a total of 300 kg ha^−1^. The amounts of N, P, and K applied as N, P_2_O_5_, and K_2_O were 267.5, 100.6, and 27.0 kg ha^−1^, respectively, during the whole growth period.

The water production function for rice is the quantitative relationship between water and rice yield. Based on previous research results, the Jensen and Minhas models, which are multiplicative models, and the Blank, Stewart, and Singh models, which are additive models, were used in the experimental design.

① Jensen model:
  $$\frac{{Y_{a} }}{{Y_{m} }} = \prod\limits_{i}^{n} {\left( {\frac{{ET_{a} }}{{ET_{m} }}} \right)}_{i}^{{\lambda_{i} }}$$
② Minhas model:
  $$\frac{{Y_{a} }}{{Y_{m} }} = \prod\limits_{i}^{n} {\left[ {1 - \left( {1 - \frac{{ET_{a} }}{{ET_{m} }}} \right)_{1}^{2} } \right]}^{{\lambda_{i} }}$$
③ Blank model:
  $$\frac{{Y_{a} }}{{Y_{m} }} = \sum\limits_{i = 1}^{n} {A_{i} } \left( {\frac{{ET_{a} }}{{ET_{m} }}} \right)_{i}$$
④ Stewart model:
  $$\frac{{Y_{a} }}{{Y_{m} }} = \sum\limits_{i = 1}^{n} {B_{i} } \left( {1 - \frac{{ET_{a} }}{{ET_{m} }}} \right)_{i}$$
⑤ Singh model:
  $$\frac{{Y_{a} }}{{Y_{m} }} = 1 - \sum\limits_{i = 1}^{n} {C_{i} } \left[ {1 - \left( {1 - \frac{{ET_{a} }}{{ET_{m} }}} \right)_{i}^{2} } \right]$$
  where *Y*_*a*_ is the actual yield under each treatment condition (kg ha^−1^); *Y*_*m*_ is the yield under normal irrigation (kg ha^−1^); *ET*_*a*_ is the actual evapotranspiration under each treatment condition (mm); *ET*_*m*_ is the evapotranspiration under normal irrigation (mm); *i* is the stage number; *n* is the total number of stages in the model; and *λ*_*i*_, *A*_*i*_, *B*_*i*_, *C*_*i*_ are the sensitivity coefficient or sensitivity index of crop yields to the lack of water.

The process of designing an optimized irrigation system is a multistage decision-making process that can be performed by a dynamic programming model.

1. Stage variables: Based on experiments building a water production function model for rice cultivated with drip irrigation under plastic film-mulched, the tillering, booting, flowering and filling stages of rice growth were taken as the research object, and *i* was the stage variable (*i* = 1, 2, 3, 4).
2. Decision variables: The decision variables were the various stages of irrigation (mi), *i* = 1, 2, 3, 4.
3. State variables: ① the amount of irrigation water available for allocation at the beginning of each growth stage (*q*_*i*_), mm, and ② the soil water storage capacity (*W*_*i*_), calculated as a function of soil moisture available to the crop in the moist layer at the beginning of stage *i*:
  $$W_{i} = 10\gamma H_{i} \left( {\overline{\theta } - \theta_{w} } \right)$$
  where *W*_*i*_ is the soil water storage available for crop utilization (mm); *γ* is the soil bulk density (g·cm^−3^); *H*_*i*_ is the plan moist layer depth in stage *i* (m); $\overline{\theta }$ is the average soil moisture content of the plan moist layer in stage *i* (%); and $\overline{\theta }$_*w*_ is the wilting coefficient.
4. The system equation describes the relationship between the variables in the state transition process, corresponding to the two-dimensional state variable.
  ① Water allocation equation:
    $$q_{i + 1} = q_{i} - m_{i}$$
    where qi and *qi* + *1* are the water supply capacity in stages *i* and *i* + *1* (mm), respectively, and *mi* is the irrigation quota in stage *i* (mm).
  ② Field water balance equation:
    $$ET = P + K + M - \left( {W_{t} - W_{0} } \right)$$
    where *ET* is the field crop water requirement within time period *t* (mm or m^3^ ha^−1^); *P* is the effective rainfall within time period *t* (mm or m^3^ ha^−1^); *K* is the groundwater recharge within time period *t* (mm or m^3^ ha^−1^); *M* is the irrigation water within time period *t* (mm or m^3^ ha^−1^); and *W*_*t*_ and *W*_*0*_ are the changes in water storage within time period *t* (mm or m^3^ ha^−1^).
5. Objective function: The Jensen model can be used as the objective function in cases where the water quantity can be allocated to the maximum yield per unit area, which is *Y*_*a*_/*Y*_*m*_ → 1.0:
  $$F = \max \left( {\frac{{Y_{a} }}{{Y_{m} }}} \right) = \max \prod\limits_{i}^{n} {\left( {\frac{{ET_{a} }}{{ET_{m} }}} \right)}_{i}^{{\lambda_{i} }}$$
  where *ET*_*mi*_ is the evapotranspiration in stage *i*, mm.
6. Constraints
  ① Irrigation constraints:
    $$0 \le m_{i} \le q_{i}$$
    $$\sum\limits_{i}^{n} {m_{i} } = Q$$
    $$0 \le ET_{ai} \le ET_{mi}$$
  ② Soil moisture constraint in the plan moist layer:
    $$\theta_{w} \le \theta_{i} \le \theta_{f} ,i = 1,2,3,4$$
    where $\overline{\theta }$_*i*_ is the average soil moisture content in the plan moist layer as a percentage of the soil dry weight and off is the water content in the field as a percentage of the soil dry weight.
  ③ the boundary constraint is the initial soil moisture constraint:
    $$\theta_{i} = \theta_{0}$$
7. Recurrence equation: The calculation is carried out through backward recursion for sequential decision-making. The recursive equation is:
  $$f_{i}^{*} \left( {q_{i} ,W_{i} } \right) = \max \left[ {R_{i} \left( {q_{i} ,m_{i} } \right) \cdot f_{i + 1}^{*} \left( {q_{i + 1} } \right)} \right],i = 1,2,3,4$$
  $$R_{i} \left( {q_{i} ,m_{i} } \right) = \left( {\frac{{ET_{ai} }}{{ET_{mi} }}} \right)_{i}^{{\lambda_{i} }} ,i = 1,2,3,4$$
  $$f_{n}^{*} \left( {q_{n} } \right) = \left( {\frac{{ET_{ai} }}{{ET_{mi} }}} \right)_{i}^{{\lambda_{i} }} ,i = 4$$
  where *R*_*i*_ (*q*_*i*_, *m*_*i*_) is the benefit of growth stage *q*_*i*_ (yuan), $f_{i + 1}^{*} \left( {q_{i + 1} } \right)$ is the maximum total benefit of the remaining growth stages (yuan), and *λ* is the sensitivity index in stage *i*.

## Results and analysis

### Effect of different water treatments on water requirements, rice yield, and water use efficiency

After three years (2018–2020), the results showed that the rice yield and water requirements decreased significantly from W1(100%θ) to W6(50–60%θ) and that the water use efficiency also decreased with the decrease in soil water content (Table 3).

**Table 3:** Effects of soil water deficit on rice yield, water requirement and water use efficiency.

Treatment NO		Water requirement (m^3^ ha^−1^)			Yield (kg ha^−1^)			Water use efficiency (kg m^−3^)		
		2018	2019	2020	2018	2019	2020	2018	2019	2020
A	W1(CK)	3875.20	4861.38	4468.13	7751.64	7245.41	8758.25	2.00	1.49	1.96
	W2	3416.00	4589.33	4229.28	7566.53	6665.86	8431.13	2.22	1.45	1.99
	W3	3311.20	4092.21	3592.82	7129.71	5950.73	7066.46	2.15	1.45	1.97
	W4	3043.20	3712.40	3359.37	6566.18	5324.88	6039.50	2.16	1.43	1.80
	W5	2682.20	3340.66	3032.99	6003.51	4213.45	5118.60	2.24	1.26	1.69
	W6	2440.20	3077.63	2802.05	4907.81	3710.26	3868.60	2.01	1.21	1.38
B	W7	3075.20	3948.61	3566.75	6986.26	5949.62	7234.55	2.27	1.51	2.03
	W8	2897.20	3817.95	3452.03	6443.30	5693.35	7127.18	2.22	1.49	2.06
	W9	2891.20	3733.01	3377.46	6295.82	5428.94	7015.22	2.18	1.45	2.08
	W10	3053.20	3799.41	3335.75	6530.92	5927.83	7179.55	2.14	1.56	2.15

The actual soil moisture content of the field was determined as a percentage of field capacity(θ), reflecting the degree of soil water deficit. In group A, WI:100% of field capacity; W2:90–100% of field capacity; W3:80–90% of field capacity; W4:70–80% of field capacity; W5:60–70% of field capacity; and W6:50–60% of field capacity. In group B, soil moisture deficit conditions were applied at different growth stages. W7: a filling stage deficit (70–80% of field capacity); W8: a flowering stage deficit (70–80%); W9: a booting stage deficit (70–80%); and W10: a tillering stage deficit (70–80%). The relative soil water content during the other growth stages was maintained at 80–90% of field capacity.

The results for group A showed that the yield decreased from 7751.6 kg ha^−1^ to 4907.8 kg ha^−1^ as the soil water content decreased fromW1 (100%θ) to W6 (50%θ) in 2018. The yields in W2 to W6 were 2.4%, 8.0%, 15.3%, 22.6% and 36.7% lower than that in W1, respectively. The water requirement decreased from 3875.2–2440.2 m^3^ ha^−1^ across the six treatments, and those in W2 to W6 were 11.9%, 14.6%, 21.5%, 30.8% and 37.0% lower, respectively, then that in treatment 1. The water use efficiency ranged between 2.00 and 2.24 kg·m^−3^. In 2019, The yield decreased from 7245.4 kg ha^−1^ to 3710.3 kg ha^−1^ as the soil water content decreased across W1 to W6. The yields of W2 to W6 were 8.0%, 17.9%, 26.5%, 41.8% and 48.8% lower than that of W1, respectively. The water requirement decreased from 4861.4–3077.6 m^3^ ha^−1^ across the six treatments, and those in W2-W6 were 5.6%, 15.8%, 23.6%, 31.3% and 36.7%, respectively, lower than that in W1. The water use efficiency decreased from 1.49–1.21 kg·m^−3^ from W1 to W6. In 2020, the yield decreased from 8758.3 kg ha^−1^ to 3868.6 kg ha^−1^ as the soil water content decreased from W1 to W6. The yields in W2 to W6 were 3.7%, 19.3%, 31.0%, 41.6% and 55.8% lower than that in W1, respectively. The water requirement decreased from 4468.1–2802.0 m^3^ ha^−1^ across the six treatments, and those in W2 to W6 were 5.4%, 19.6%, 24.8%, 32.1% and 37.3% lower, respectively, then that in W1. The water use efficiency decreased from 1.99–1.38 kg·m^−3^ across the six treatments.

The results from group B showed that from W7 to W10, the relative water content of the soil remained at 80–90% of the field capacity but decreased by 10% in the different deficit growth stages and that the rice yield and water demand decreased to different degrees. In 2018, compared with that in W1, the rice yields in W7-W10 decreased by 9.9%, 16.9%, 18.8%, and 15.7%, respectively. The corresponding water demand decreased by 20.6%, 25.2%, 25.4%, and 21.2%, respectively. The water use efficiency ranged between 2.27–2.14 kg·m^−3^. In 2019, compared with that in W1, the rice yields in W7-W10 decreased by 17.9%, 21.4%, 25.1%, and 18.2%, respectively. The corresponding water demand decreased by 18.8%, 21.5%, 23.2%, and 21.9%, respectively. The water use efficiency ranged between 1.45–1.56 kg m^−3^. In 2020, compared with that in W1, the rice yields in W7-W10 decreased by 17.4%, 18.6%, 19.9%, and 18.0%, respectively. The corresponding water demand decreased by 20.2%, 22.7%, 24.4%, and 25.3%, respectively. The water use efficiency ranged between 1.45–1.56 kg·m^−3^. The results show that the strength of the effect of water deficit on yield decreased in the order booting stage > flowering stage > tillering stage > filling stage.

The results of the variance analysis for the 2018–2020 experiment show that the average rice yield was significantly different among treatments (F = 29.387 > F0.01 = 5.636) and that there were also significant differences among the years (F = 11.080 > F0.01 = 7.559).

The results of multiple comparisons for group A (Table 4) showed that there was no significant difference in yield between W1 and W2; W1 and W 2 differed significantly from W3, W4, W5 and W6; and W6 had the lowest yield. There were significant differences in yield (F = 72.161 > F0.01 = 5.636) between crop treatments, and significant differences in water requirements were also found among the treatments (F = 62.565 > F0.01 = 7.559). The water requirement of W1 was significantly different from those of W3, W4, W5 and W6, and W6 had the lowest water requirement. Comparing the 3 years, the average yield of all treatments was 6654.2, 5518.4, and 6547.1 kg ha^−1^; the average water requirements were 3128.0, 3945.6, and 3580.8 m^3^ ha^−1^; and the water use efficiency was 2.13, 1.38 and 1.80 kg·m^−3^ in 2018, 2019 and 2020, respectively. The multiple comparisons results showed that the yield and water use efficiency in 2018 and 2020 were significantly higher than those in 2019 and that the water requirements in 2018 and 2020 were significantly lower than that in 2019. On average over the three years, with drip irrigation under plastic film-mulched, when the soil moisture content changed from 100 to 90% of field capacity, the rice yield and water demand were not significantly reduced (average decrease of only 4.7%), the water requirements decreased by 7.6%, and the water use efficiency was relatively high. Therefore, W2 could be used as an indicator of appropriate irrigation quotas and irrigation quantities for rice, considering yield differences, yield reductions and water use efficiency; such quotas could reduce save water resource use. W3-W6, due to the soil water deficit, would result in significant yield reductions and low water use efficiency and are therefore not recommended as production practices. However, a high-water supply (such as inW1) does not necessarily result in high water use efficiency in rice. Although higher yields can be obtained, the water demand is also high, resulting in low water use efficiency. As shown by W2, maintaining rice at 90% of field capacity with irrigation can result in high yields and high efficiency at the same time.

**Table 4:** Significance of differences of both rice grain yield and water requirement under soil water deficit (Duncan).

Treatment NO		Yield (kg ha^−1^)	Water requirement (m^3^ ha^−1^)	Water use efficiency (kg m^−3^)
A	W1	7918.4 ± 444.61a	4401.6 ± 286.62a	1.82 ± 0.162
	W2	7554.5 ± 509.62a	4078.2 ± 347.03ab	1.89 ± 0.226
	W3	6715.6 ± 382.89ab	3665.4 ± 228.36abc	1.86 ± 0.209
	W4	5976.8 ± 359.70abc	3371.7 ± 193.28bcd	1.80 ± 0.209
	W5	5111.8 ± 516.75bc	3018.6 ± 190.22bcd	1.73 ± 0.283
	W6	4162.2 ± 375.58ab	2773.3 ± 184.57bcd	1.53 ± 0.245

The actual soil moisture content of the field was determined as a percentage of field capacity(θ), reflecting the degree of soil water deficit. In group A, WI:100% of field capacity; W2:90–100% of field capacity; W3:80–90% of field capacity; W4:70–80% of field capacity; W5:60–70% of field capacity; and W6:50–60% of field capacity. In group B, soil moisture deficit conditions were applied at different growth stages. W7: a filling stage deficit (70–80% of field capacity); W8: a flowering stage deficit (70–80%); W9: a booting stage deficit (70–80%); and W10: a tillering stage deficit (70–80%). The relative soil water content during the other growth stages was maintained at 80–90% of field capacity.

Data in the table was the average and standard error of 3 years. Different letters after data in the table mean significant at 5% level.

The results of multiple comparisons in group B (Table 5) and the variance analysis for W1, W2, W7, W8, W9 and W10 showed that the rice yield was significantly different (F = 57.829 > F0.01 = 5.636) among the different treatments and that there were also significant differences among the years (F = 137.746 > F0.01 = 7.559). The 3a average yield reduction showed that the water deficit at different growth stages had a certain effect on the yield, and the degree of the effect decreased in the order booting stage > flowering stage > tillering stage > filling stage. There was no significant difference in yield between W7, W8, W9 and W10, but their yields were all significantly lower than that of W1. In contrast, W9 (water deficit at the booting stage) had a significant effect on yield, with an average yield reduction of 21.3%, a lower water requirement, and lower water use efficiency than the other treatments. W8 and W10 (water deficits at the flowering stage and tillering stage, respectively) also affected yield levels, with yield reductions of 19.0% and 17.3%, respectively. W7 (water deficit at the filling stage) had a relatively small effect on yield, with an average yield reduction of 15.1%. Thus, when the soil moisture content is maintained at 80–90% of field capacity, the relative soil water content decreases by 10% at different growth stages, which results in a decrease in rice yield and a certain difference among the different growth stages. The degree of influence was as follows: booting stage > flowering stage > tillering stage > filling stage.

**Table 5:** Significance of differences of both rice grain yield and water requirement under conditions of soil water deficit (Duncan).

Treatment NO		Yield (kg ha^−1^)	Water requirement (m^3^ ha^−1^)	Water use efficiency (kg m^−3^)
B	W1	7918.4 ± 444.61a	4401.6 ± 286.62a	1.82 ± 0.164
	W2	7554.5 ± 509.62ab	4078.2 ± 347.03ab	1.89 ± 0.226
	W7	6723.5 ± 393.51bc	3530.2 ± 252.79ab	1.94 ± 0.226
	W8	6421.3 ± 414.06bcd	3389.1 ± 267.66b	1.93 ± 0.223
	W9	6246.7 ± 458.58 cd	3333.9 ± 243.98b	1.90 ± 0.226
	W10	6546.1 ± 361.42bcd	3396.1 ± 217.52b	1.95 ± 0.195

The actual soil moisture content of the field was determined as a percentage of field capacity(θ), reflecting the degree of soil water deficit. In group A, WI:100% of field capacity; W2:90–100% of field capacity; W3:80–90% of field capacity; W4:70–80% of field capacity; W5:60–70% of field capacity; and W6:50–60% of field capacity. In group B, soil moisture deficit conditions were applied at different growth stages. W7: a filling stage deficit (70–80% of field capacity); W8: a flowering stage deficit (70–80%); W9: a booting stage deficit (70–80%); and W10: a tillering stage deficit (70–80%). The relative soil water content during the other growth stages was maintained at 80–90% of field capacity.

Data in the table was the average and standard error of 3 years. Different letters after data in the table mean significant at 5% level.

### Relationship between rice production and total water demand

**Figure 2:**
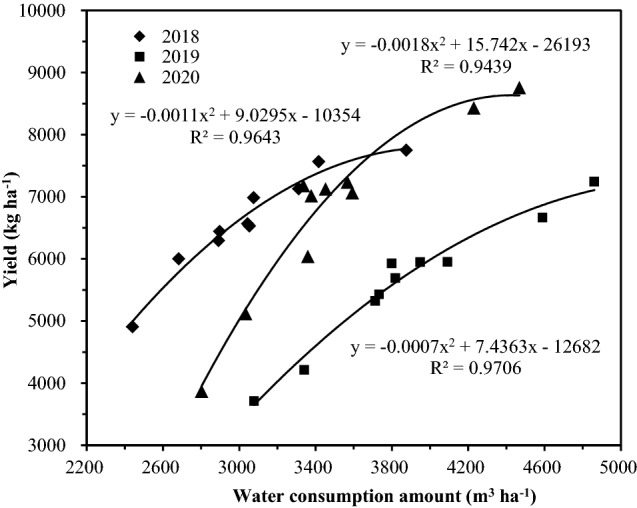
Relationships between rice grain yield under dry-farming and water requirement (2012–2014).

The results of the 3-year experiment were plotted as a scatter plot (Fig. 2).

The results (Fig. 2) show that there is a parabolic relationship between rice water requirements and yield. After regression analysis and curve selection, the rice water requirement (*X*) and rice yield (*Y*) was described with a quadratic function (*y* = *ax*^*2*^ + *bx* + *c*). The resulting regression equation was calculated, and the highest water demand and the highest yield value were calculated (Table 6).

**Table 6:** Regression equations of both rice grain yield under dry-farming and water requirement (2018–2020).

Years	Regression equation	Determination coefficient (R^2^)	Water requirement in highest yield (m^3^ ha^−1^)	Highest yield (kg ha^−1^)
2018	y = −0.00112300x^2^ + 9.02952860x − 10353.7800	0.9643**	4020.3	7796.8
2019	y = −0.00069228x^2^ + 7.43625435x − 12681.6534	0.9706**	5370.8	7387.8
2020	y = −0.00177873x^2^ + 15.74168978x − 26193.3419	0.9439**	4425.0	8635.0
Average	y = −0.00116240x^2^ + 10.59355145x − 16266.8605	0.9799**	4556.8	7869.3

*, **Means significant at 5% and 1%, respectively.

The results (Table 6) show that there is a significant positive correlation (r > r0.01) between the water requirements of rice cultivated with drip irrigation under plastic film-mulched and yield. The rice yields in 2018–2020 plotted against the water requirement can be described with a quadratic function. The highest water demand and its highest yield were calculated by this equation. The highest water demands in 2018, 2019 and 2020 were 4020.3, 5370.8 and 4425.0 m^3^ ha^−1^, respectively. The highest yields were 7796.8, 7387.8 and 8635.0 kg ha^−1^, respectively. The 3a regression equation was obtained by using the 3a water demand and the production average allocation equation. The average water demand was 4556.8 m^3^ ha^−1^, and the highest yield was 7869.3 kg ha^−1^. When the water demand for rice was lower than that associated with the highest yield, the yield of rice increased with increasing in water demand. When the water requirement of the growth period was higher than the highest water requirement, the yields decreased. Therefore, in the practice of rice production, it is necessary to consider the water requirements for normal rice growth and development as well as water-saving practices and high yields.

### Water production function model for rice cultivated with drip irrigation under plastic film-mulched

According to the experimental results obtained by applying different soil water conditions at different growth stages, the tested models were mathematically transformed into multivariate linear equations, and the multivariate linear equations were solved with the least-squares method. The exponents of the different models and the values of the coefficients *λ*_*i*_, *A*_*i*_, *B*_*i*_, and *C*_*i*_ were obtained (Table 7).

**Table 7:** Sensitive coefficient or sensitivity index in the water production function models.

Years	*λ* in Jensen model			
	①Tillering stage	②Booting stage	③Flowering stage	④Filling stage
2018	0.1644	0.4983	0.2245	0.1087
2019	0.0320	0.3620	0.2843	0.0112
2020	0.1284	0.4529	0.2439	0.0641
Average	0.1083	0.4377	0.2509	0.0613

Years	*λ* in Minhas model			
	①Tillering stage	②Booting stage	③Flowering stage	④Filling stage
2018	0.2014	0.5478	0.3574	0.0647
2019	0.3004	0.4857	0.3711	0.1248
2020	0.1756	0.5712	0.2547	0.0958
Average	0.2258	0.5349	0.3277	0.0951

Years	*A* in Blank model			
	①Tillering stage	②Booting stage	③Flowering stage	④Filling stage
2018	0.1248	0.6471	0.3214	0.0145
2019	0.1647	0.5749	0.4251	0.0695
2020	0.1752	0.5135	0.4185	0.0713
Average	0.1549	0.5785	0.3883	0.0518

Years	*B* in Stewart model			
	①Tillering stage	②Booting stage	③Flowering stage	④Filling stage
2018	0.0632	0.2147	0.1542	0.0062
2019	0.0623	0.2359	0.1846	0.0124
2020	0.0471	0.1987	0.1102	0.0046
Average	0.0575	0.2164	0.1497	0.0077

Years	*C* in Singh model			
	①Tillering stage	②Booting stage	③Flowering stage	④Filling stage
2018	0.4685	0.9651	1.0587	2.1459
2019	0.2479	0.7451	1.4789	2.8745
2020	0.2597	0.4985	1.0201	3.5412
Average	0.3254	0.7362	1.1859	2.8539

The water production function for rice is the quantitative relationship between water and rice yield. Based on previous research results, the Jensen and Minhas models, which are multiplicative models, and the Blank, Stewart, and Singh models, which are additive models, were used in the experimental design. And λi, Ai, Bi, Ci are the sensitivity coefficient or sensitivity index of crop yields to the lack of water.

Table 7 shows that:

1. In the Jensen model, the order of *λ* values from high to low in the different growth stages was ② > ③ > ① > ④. The larger the value of *λ* is, the smaller the *Y*_*a*_/*Y*_*m*_ value is after a water shortage, which means that *λ* is more sensitive to water shortages. The value of *λ* was sensitive to water, which is consistent with the physiological characteristics of rice and the results of the irrigation experiments. Therefore, the Jensen model is a reasonable rice water production function for this region.
2. In the Minhas model, the order of *λ* values from high to low was ② > ③ > ① > ④. The larger the value of *λ* is, the smaller the *Y*_*a*_*/Y*_*m*_ value is after a water shortage, which means that λ is more sensitive to water shortages. The value of *λ* was sensitive to water, which is consistent with the physiological characteristics of rice and the results of the irrigation experiments. Therefore, the Minhas model is a reasonable rice water production function for this region.
3. In the Blank model, the order of *A* values from high to low was ② > ③ > ① > ④. The larger the value of *A* is, the higher the *Y*_*a*_*/Y*_*m*_ value is after a water shortage, which means that it is not sensitive to water shortages. The value of *A* was not sensitive to water but was consistent with the physiological characteristics of rice and the results of the irrigation experiments. Therefore, the Blank model is not a reasonable rice water production function for this region.
4. In the Stewart model, the order of *B* values from high to low was ② > ③ > ① > ④. The larger the value of *B* is, the smaller the *Y*_*a*_*/Y*_*m*_ value is after a water shortage, which means that it is more sensitive to water shortage. The value of *B* was sensitive to water, which is consistent with the physiological characteristics of rice and the results of the irrigation experiments. Therefore, the Stewart model is a reasonable rice water production function for this region.
5. In the Singh model, the order of *C* values from high to low was ④ > ③ > ② > ①. The smaller the value of *C* is, the smaller the *Y*_*a*_*/Y*_*m*_ value is after a water shortage, which means that it is more sensitive to water shortage. However, the peak in the ④ stage is inconsistent with the irrigation experimental results and the physiological characteristics of rice. Thus, the Singh model is not a reasonable rice water production function for this region.

Therefore, the Jensen, Minhas, and Stewart models were used to generate water production functions for rice in Ningxia. The results were as follows:

Jensen model:

$$\frac{{Y_{a} }}{{Y_{m} }} = \left( {\frac{{ET_{a} }}{{ET_{m} }}} \right)_{1}^{0.1083} \cdot \left( {\frac{{ET_{a} }}{{ET_{m} }}} \right)_{2}^{0.4377} \cdot \left( {\frac{{ET_{a} }}{{ET_{m} }}} \right)_{3}^{0.2509} \cdot \left( {\frac{{ET_{a} }}{{ET_{m} }}} \right)_{4}^{0.0613}$$

Minhas model:

$$\frac{{Y_{a} }}{{Y_{m} }} = \left[ {1 - \left( {1 - \frac{{ET_{a} }}{{ET_{m} }}} \right)_{1}^{2} } \right]^{0.2258} \cdot \left[ {1 - \left( {1 - \frac{{ET_{a} }}{{ET_{m} }}} \right)_{2}^{2} } \right]^{0.5349} \cdot \left[ {1 - \left( {1 - \frac{{ET_{a} }}{{ET_{m} }}} \right)_{3}^{2} } \right]^{0.3277} \cdot \left[ {1 - \left( {1 - \frac{{ET_{a} }}{{ET_{m} }}} \right)_{4}^{2} } \right]^{0.0951}$$

Stewart model:

$$\frac{{Y_{a} }}{{Y_{m} }} = 0.0575\left( {1 - \frac{{ET_{a} }}{{ET_{m} }}} \right)_{1} \cdot 0.2146\left( {1 - \frac{{ET_{a} }}{{ET_{m} }}} \right)_{1} \cdot 0.1497\left( {1 - \frac{{ET_{a} }}{{ET_{m} }}} \right)_{1} \cdot 0.0077\left( {1 - \frac{{ET_{a} }}{{ET_{m} }}} \right)_{1}$$

### Optimal irrigation schedule for rice cultivated with drip irrigation under plastic film

Based on the water production function model obtained herein, we designed an optimum irrigation schedule for drip irrigation under plastic film (Table 8). The design of such an irrigation schedule can have a unit of time of one week, ten days or the growth stage. To correspond with the water production function model, the irrigation system model was designed with growth stage as the unit. The whole growth period was divided into 4 stages (Tillering stage, booting stage, flowering stage, and filling stage). Combined with the basic parameters of the irrigation system designed for rice cultivated with drip irrigation under plastic film, the optimum irrigation schedule was obtained by developing a computer program that could consider the critical period of rice crop water requirements.

**Table 8:** Basic parameters of irrigation system design.

Growth stage	Tillering stage	Booting stage	Flowering stage	Filling stage
*ET*_m_/(m^3^ ha^−1^)	755.82	1895.82	1226.82	523.12
*λ*	0.1083	0.4377	0.2509	0.0613

Table 9 shows that with the increase in rice production under drip irrigation, the water supply capacity significantly increased. When the water supply capacity was 2000 m^3^ ha^−1^, the yield was 52.6% of that under full irrigation; when the water supply capacity was 2500 m^3^ ha^−1^, the yield was 62.6% of that under full irrigation; when the water supply capacity was 3000 m^3^ ha^−1^, the yield was 73.1% of that under full irrigation; when the water supply was 3500 m^3^ ha^−1^, the yield was 83.3% of that under full irrigation; when the water supply was 4000 m^3^ ha^−1^, the yield was 93.2% of that under full irrigation; when the water supply capacity was 4500 m^3^ ha^−1^, the yield was 103.0% of that under full irrigation; when the water supply capacity was 5000 m^3^ ha^−1^, the yield was 112.5% of that under full irrigation; and when the water supply capacity was 5500 m^3^ ha^−1^, the yield was 122.0% of that under full irrigation. As the water supply capacity increases, the consequent increases in yield become less noticeable. The water supply range that produced the most obvious yield increases was 4000–4500 m^3^ ha^−1^, and the optimum yield would be produced when the water supply reached 4250 m^3^ ha^−1^. The optimal irrigation schedule was 750 m^3^ ha^−1^ at the tillering stage, 2125 m^3^ ha^−1^ at the booting stage, 1050 m^3^ ha^−1^ at the flowering stage, and 325 m^3^ ha^−1^ at the filling stage.

**Table 9:** Irrigation system optimization of rice under drip irrigation.

Available water (m^3^ ha^−1^)	Irrigation amount (m^3^ ha^−1^)				*Y*_*a*_*/Y*_*m*_
	Tillering stage	Booting stage	Flowering stage	Filling stage	
2000	300	1000	600	100	0.5163
2500	400	1250	700	150	0.6259
3000	500	1500	800	200	0.7309
3500	600	1750	900	250	0.8327
4000	700	2000	1000	300	0.9321
4500	800	2250	1100	350	1.0295
5000	900	2500	1200	400	1.1252
5500	1000	2750	1300	450	1.2195

## Discussion

Over-irrigation of rice can lead to excessive vegetative growth and it can also cause leaching of nutrients out of the root zone, increasing fertilizer costs and contaminating groundwater supplies^24–26^. At present, many domestic and foreign scholars have carried out theoretical research on the optimal irrigation schedule of deficit irrigation^27–29^. Crop yields are affected not only by the total irrigation volume but also by the distribution of the total irrigation volume among the different crop growth stages^30^. At present, Jensen's crop water production function is used as the objective function^31,32^, and irrigation optimization is performed based on the soil water content and water supply capacity to optimize irrigation systems regarding the final total output and provincial water savings^33,34^. In this study, the irrigation system for rice cultivated with drip irrigation under plastic film was optimized based on the Jensen model. Experiments related to the water production function of rice cultivated with drip irrigation under plastic film revealed that different levels of water deficit as well as water deficit conditions at different growth stages had significant effects on rice production. The results show that the yield under water deficit conditions at 50% of field capacity was significantly lower than that at 100% of field capacity. Water deficit at the booting stage had the strongest negative effect on the final yield of rice. The water requirements of rice changed among its growth stages in the order booting stage > flowering stage > tillering stage > filling stage. The results of the regression analysis of the total water requirement and the total yield of drip-irrigated rice showed that the yield-water demand relationship formed a quadratic parabola. The peak values of water demand were 4104.32 m^3^ ha^−1^, 5311.64 m^3^ ha^−1^, and 4372.69 m^3^ ha^−1^, and the maximum yields reached 8176.19 kg ha^−1^, 7167.83 kg ha^−1^ and 8223.48 kg ha^−1^, respectively, in 2018, 2019 and 2020. When the total water demand was lower than the peak value, the yield of rice increased with increasing water demand. When the water requirement was greater than the peak value, the yield decreased with increasing water demand^34^. Based on this model, water sensitivity indices were obtained from five models, and three water production function models suitable for drip irrigation for rice in the study area were established. Based on these models, it was found that the effect of yield became increasingly obvious with the increasing amount of water supplied.

## Conclusions

Experiments related to the water production function of rice cultivated with drip irrigation under plastic film revealed that different levels of water deficit as well as water deficit conditions at different growth stages had significant effects on rice production. The results show that:

1. the yield under water deficit conditions at 50% of field capacity was significantly lower than that at 100% of field capacity. Water deficit at the Booting stage had the strongest negative effect on the final yield of rice.
2. The water requirements of rice changed among its growth stages in the order: booting stage > flowering stage > tillering stage > filling stage.
3. The results of the regression analysis of the total water requirement and the total yield of drip-irrigated rice showed that the yield-water demand relationship formed a quadratic parabola. The peak values of water demand were 4104.32 m^3^ ha^−1^, 5311.64 m^3^ ha^−1^, and 4372.69 m^3^ ha^−1^, and the maximum yields reached 8176.19 kg ha^−1^, 7167.83 kg ha^−1^ and 8223.48 kg ha^−1^, respectively, in 2018, 2019 and 2020.
4. The water supply range that produced the most notable yield increases was between 4000–4500 m^3^ ha^−1^. The optimal yield can be achieved with a water supply of 4250 m^3^ ha^−1^. The optimal irrigation schedule was 750 m^3^ ha^−1^ at the tillering stage, 2125 m^3^ ha^−1^ at the booting stage, 1050 m^3^ ha^−1^ at the flowering stage, and 325 m^3^ ha^−1^ at the filling stage.

The conclusions of this study can provide a technical theoretical basis for implementing and popularizing rice cultivation with drip irrigation in Ningxia China.

## Data Availability

The datasets generated and analysed during the current study are not publicly available due to some of the data will have implications for further research, but are available from the corresponding author on reasonable request.

## References

[1] Detar WR (2008). Yield and growth characteristics for cotton under various irrigation regimes on sandy soil. Agric. Water Manag..

[2] Abdelaziz, H., *et al*. The combined effect of deficit irrigation by treated wastewater and organic amendment on quinoa (Chenopodium quinoa Willd.) productivity. *Desalin. Water Treatment* **52**(10–12), 2208–2213. (2013).

[3] Pereira LS, Oweis T, Zairi A (2002). Irrigation management under water scarcity. Agric. Water Manag..

[4] Ogola JBO, Wheeler TR, Harris PM (2002). Effects of nitrogen and irrigation on water use of maize crops. Field Crops Res..

[5] Zhang Y, Kendy E, Yu Q, Liu C, Shen Y, Sun H (2004). Effect of soil water deficit on evapotranspiration, crop yield, and water use efficiency in the north china plain. Agric. Water Manag..

[6] Wang, D.Y., Xu, C.M., &Yuan, J. Changes in agronomic traits of Indica hybrid rice during genetic improvement. *Agric. Sci. Technol.* **12**(8), 1146–1152 (2011).

[7] Zhang, X., & Qiu, G.. Causes of excessive use of chemical fertilizer and its impacts on China′s water environment security. South-to-North Water Transfers and Water Science & Technology (2019).

[8] Xu, G. A Controllable irrigation technology of water-saving and high-yield. *China Rural Nater & Hydropower* (2017).

[9] Zhang F, Li M, Qi J (2015). Plastic film mulching increases soil respiration in ridge-furrow Maize Management. Arid Soil Res. Rehabil..

[10] Gao, X., Hoffland, E., & Stomph, T. J. Improving zinc bioavailability in transition from flooded to aerobic rice. A review. *Agron. Sustain. Dev.* **32**(2), 465–478 (2012).

[11] Mahmoudzadeh VM (2016). Crop water production functions—A review of available mathematical method. J. Agric. Sci..

[12] Ferrers E, Soriano MA (2007). Deficit irrigation for reducing agricultural water use. J. Exp. Bot..

[13] Zhang BC, Li FM, Huang GB, Cheng ZY, Zhang YH (2006). Yield performance of spring wheat improved by regulated deficit irrigation in an arid area. Agric. Water Manag..

[14] Cui N, Du T, Kang S, Li F, Zhang J, Wang M (2008). Regulated deficit irrigation improved fruit quality and water use efficiency of pear-jujube trees. Agric. Water Manag..

[15] Zhang HP, Oweis T (1999). Water–yield relations and optimal irrigation scheduling of wheat in the Mediterranean region. Agric. Water Manag..

[16] Zwart SJ, Bastiaanssen W (2004). Review of measured crop water productivity values for irrigated wheat, rice, cotton and maize. Agric. Water Manag..

[17] Oktem A (2008). Effect of water shortage on yield, and protein and mineral compositions of drip-irrigated sweet corn in sustainable agricultural systems. Agric. Water Manag..

[18] Yuan BZ, Kang Y, Nishiyama S (2001). Drip irrigation scheduling for tomatoes in unheated greenhouses. Irrigat. Sci..

[19] Ibragimov N, Evett SR, Esanbekov Y, Kamilov BS, Mirzaev L, Lamers JPA (2007). Water use efficiency of irrigated cotton in Uzbekistan under drip and furrow irrigation. Agric. Water Manag..

[20] Horst MG, Shamutalov SS, Gonçalves JM, Pereira LS (2007). Assessing impacts of surge-flow irrigation on water saving and productivity of cotton. Agric. Water Manag..

[21] Wang Y, Li M, Lan M (2011). Effect of soil wetting pattern on cotton-root distribution and plant growth under plastic mulched drip irrigation in field. Trans. Chin. Soc. Agric. Eng..

[22] Dagdelen, N., Basal, H., Yilmaz, E., T Gürbüz, & Akay, S. Different drip irrigation regimes affect cotton yield, water use efficiency and fiber quality in western turkey. *Agric. Water Manag.* **96**(1), 111–120 (2009).

[23] Cheng WG, Lu WX, Zhang Z, Chu H (2016). Adaptability of various models of the water production function for rice in Jilin province, china. Paddy Water Environ..

[24] Wang, Y. T., *et al*. Fertilizer source and medium composition affect vegetative growth and mineral nutrition of a hybrid moth orchid. *J. Am. Soc. Hortic. Sci.* (2002).

[25] Imran, M., & Gurmani, Z. A. Role of macro and micro nutrients in the plant growth and development. *Sci. Technol. Dev.* (2011).

[26] Jo, S.-G., Kang, Y.-I., Om, K.-S., Cha, Y.-H., Ri, S.-Y. Growth, photosynthesis and yield of soybean in ridge-furrow intercropping system of soybean and flax. *Field Crops Res.* **275 **(2022).

[27] Li D, Wang X (2021). Assessing irrigated water utilization to optimize irrigation schedule in the oasis-desert ecotone of Hexi Corridor of China. Agric. Ecosyst. Environ..

[28] Cheng, G., Shi, H., Ruiping, L. I., *et al*. Research on the Optimal Irrigation Schedule of Semi-fixed Sprinkler Soybean Based on the ISAREG Model. *J. Irrigat. Drainag*. (2015).

[29] Akhtar F, Tischbein B, Awan UK (2012). Optimizing deficit irrigation scheduling under shallow groundwater conditions in lower reaches of Amu Darya River Basin. Water Resour. Manag..

[30] Jinyu He, Juncang T (2015). Model of coupling water with fertilizer and optimum combination scheme of rice cultivated in aerobic soil with drip irrigation under plastic film. Trans. Chin. Soc. Agric. Eng..

[31] Shun-Jun, H. U. Cumulative function of sensitive index of Jensen's crop water production model for cotton. *J. Shenyang Agric. Univ.* (2004).

[32] Chen X, Duan C, Lin Q (2008). Application of large-scale system model based on particle swarm optimization to optimal allocation of water resources in irrigation areas. Trans. Chin. Soc. Agric. Eng..

[33] Dai ZY, Li YP (2013). A multistage irrigation water allocation model for agricultural land-use planning under uncertainty. Agric. Water Manag..

[34] Dabach S, Lazarovitch N (2013). Numerical investigation of irrigation scheduling based on soil water status. Irrig. Sci..

